# Is it time to recommend AUC-based vancomycin therapeutic drug monitoring only? A cross-sectional survey in China

**DOI:** 10.3389/fphar.2024.1370040

**Published:** 2024-07-12

**Authors:** Jieqiong Liu, Xuan Zhang, Gang Liang, Jianping Zhu, Yi Yang, Ying Zheng, Yun Han, Lingyan Yu, Yuhua Zhao, Zhenwei Yu

**Affiliations:** ^1^ Sir Run Run Shaw Hospital, Zhejiang University School of Medicine, Hangzhou, China; ^2^ The 903rd Hospital of PLA Joint Logistic Support Force, Hangzhou, China; ^3^ Northern Jiangsu People’s Hospital, Yangzhou, China; ^4^ College of Pharmaceutical Science, Zhejiang University, Hangzhou, China; ^5^ Research Center for Clinical Pharmacy, Zhejiang University, Hangzhou, China; ^6^ The Second Affiliated Hospital, Zhejiang University School of Medicine, Hangzhou, China; ^7^ Affiliated Xiaoshan Hospital, Hangzhou Normal University, Hangzhou, China

**Keywords:** vancomycin, survey, therapeutic drug monitoring, trough concentration, area under the concentration-time curve

## Abstract

**Background:**

The latest published therapeutic drug monitoring (TDM) guidelines for vancomycin recommend changing trough-based monitoring to area under the concentration-to-time curve (AUC)-based monitoring. This study aimed to evaluate the implementation status and perceptions of vancomycin AUC-based TDM in China and to determine the challenges in performing AUC-based TDM.

**Methods:**

A nationwide cross-sectional survey was conducted in China using an online questionnaire. The questionnaire comprised a total of 25 questions with open- and closed-ended answers to collect information about the current implementation of vancomycin TDM and the participants’ perceptions of these practices. The questionnaire responses were collected via the Questionnaire Star platform and analyzed.

**Results:**

A total of 161 questionnaires were completed by 131 hospitals and were included. Approximately 59.5% (78/131) of the surveyed hospitals conducted vancomycin TDM; however, only 10.7% (14/131) of these hospitals performed AUC-based vancomycin TDM. Of the eligible participants, 58.4% (94/161) had experience with vancomycin TDM, and only 37 participants (37/161, 23.0%) had the ability to estimate the AUC, primarily through Bayesian simulation (33/161, 20.5%). The participants considered the following challenges to implementing AUC-based monitoring: (1) the high cost of AUC-based monitoring; (2) inadequate knowledge among pharmacists and/or physicians; (3) the complexity of AUC calculations; (4) difficulty obtaining AUC software; and (5) unclear benefit of AUC-based monitoring.

**Conclusion:**

The majority of surveyed hospitals have not yet implemented AUC-based vancomycin TDM. Multiple challenges should be addressed before wide implementation of AUC-based monitoring, and guidance for trough-based monitoring is still needed.

## Introduction

Vancomycin is a commonly used glycopeptide antibiotic in clinical practice for the treatment of serious infections caused by gram-positive bacteria, including methicillin-resistant *Staphylococcus aureus* (MRSA) (Tong et al., 2015; Burns and Goldman, 2020). Vancomycin has a narrow therapeutic window and large interindividual pharmacokinetic variability; thus, therapeutic drug monitoring (TDM) has been a key approach for maximizing its therapeutic efficacy and minimizing the risk of nephrotoxicity (Perin et al., 2020). The optimal TDM practice for vancomycin is evolving but still controversial (Jorgensen et al., 2021; Lodise and Drusano, 2021). The 2009 American guideline recommends monitoring vancomycin trough concentrations in routine clinical practice, which can be used as a surrogate marker for the 24-hour area under the curve (AUC) because of the historical difficulty in estimating the AUC for vancomycin (Rybak et al., 2009). This guideline recommended a target trough concentration of 15–20 mg/L to increase the likelihood of attaining an AUC of ≥400 mg h/L (Rybak et al., 2009). However, there is increasing evidence of limitations in vancomycin trough monitoring, such as a poor linear relationship between trough concentrations and the AUC, and that trough-guided TDM possibly leads to overexposure, thereby increasing the risk of nephrotoxicity (Patel et al., 2011; Clark et al., 2019; Yamada et al., 2023). In light of these findings and the increasing accessibility of AUC estimation software, the 2020 American guideline and 2022 Japanese guideline recommended a pivotal change in vancomycin TDM target from trough to 24-hour area under the curve/minimum inhibitory concentration (AUC/MIC) or AUC (with a surrogate MIC of 1 mg/L), which is in accordance with its pharmacokinetics and pharmacodynamics profile and no longer recommended the trough guided doing (Rybak et al., 2020; Matsumoto et al., 2022). As it would be a challenge for pharmacists and physicians to estimate the AUC based on limited samples in routine clinical practice, the Chinese guideline recommended the AUC and trough concentration both for vancomycin TDM (He et al., 2020).

Currently, there is limited knowledge regarding the implementation status of AUC-based vancomycin TDM in Chinese hospitals, as well as a lack of understanding about the perceptions of pharmacists and physicians regarding AUC-guided vancomycin monitoring. Thus, we conducted this nationwide cross-sectional survey to determine the overall implementation status, perception and knowledge of AUC-based vancomycin monitoring and to identify the main difficulties in performing AUC-based TDM. The findings of this study will provide valuable evidence for determining the current extent and approach to implementing vancomycin AUC-based monitoring and provide guidance on how to further implement vancomycin monitoring in the future.

## Methods

### Study design

This nationwide cross-sectional survey was conducted in China using an online questionnaire. A convenient sampling approach was applied to enroll participants throughout mainland China in August 2023. The participants were invited to answer the questions through a link to the questionnaire via social media (WeChat group). Participation was voluntary, confidential, and anonymous.

The ethics committee of Sir Run Shaw Hospital, School of Medicine, Zhejiang University, reviewed the protocol and decided that ethical approval was not needed.

### Questionnaire development and data collection

The questionnaire comprised a total of 25 questions with open- and closed-ended answers to collect information about the current implementation status of vancomycin TDM and the participants’ perceptions of these practices. The English version of the questionnaire is available in Supplementary Table S1. This survey was created by investigators, and the questionnaire piloting was conducted by several anti-infective clinical pharmacists to assess its relevance, clarity, validity, reliability and completeness. The data collected in the survey included the participants’ demographic information, the implementation status of vancomycin TDM in the participants’ hospitals, the pattern of vancomycin TDM (e.g., trough-based TDM or AUC-based TDM), the participant’s ability to estimate the AUC of vancomycin, the method of estimating the AUC of vancomycin (e.g., Bayesian estimation or first-order PK equations), and the participants’ perceptions about changing the vancomycin TDM strategy from trough-based to AUC-based and challenges or barriers to implementing AUC-based vancomycin TDM. This questionnaire was designed with skip logic to reduce the completion time and minimize survey fatigue.

The questionnaire responses were collected via the Questionnaire Star platform (https://www.wjx.cn/), which is the largest online survey platform in China, and analyzed via Microsoft Excel 2019 (Yin et al., 2022). When “other” answers were selected for certain questions, the investigators independently reviewed the free-text responses and assessed the intent of their responses. Based on the investigators’ assessments, responses with similar intent were classified together. All the results are presented descriptively as numbers and percentages.

## Results

### Participant characteristics

A total of 162 questionnaire responses were obtained from 131 hospitals in 20 provinces in China. One questionnaire was excluded from the final analysis because of an incomplete response. Therefore, 161 participants with complete responses were eligible and included in the analysis. The demographic characteristics of the participants and hospitals are shown in Tables 1, 2. The main participants were pharmacists from tertiary hospitals.

**TABLE 1:** Demographic characteristics of the participants.

Variable	Total (n = 161)
Department	
Pharmacy	138 (85.7%)
ICU	18 (11.2%)
Emergency medicine	2 (1.24%)
Others	3 (1.86%)
Position	
Pharmacist	141 (87.6%)
Physician	20 (12.4%)
Areas of specialization	
Respiratory	21 (13.0%)
Infectious diseases	49 (30.4%)
ICU	39 (24.2%)
Hematology	3 (1.86%)
General	15 (9.32%)
Others	65 (40.4%)
Experience in working	
1–3 years	17 (10.6%)
4–6 years	17 (10.6%)
7–9 years	30 (18.6%)
≥10 years	96 (59.6%)
Experience of vancomycin TDM	94 (58.4%)
Estimation AUC	
Available	37 (23.0%)

Abbreviations: ICU, Intensive Care Unit; TDM, therapeutic drug monitoring; AUC, 24-h area under the curve.

**TABLE 2:** Hospital characteristics of the surveyed medical centers.

Variable	Total (n = 131)
Region	
East	93 (71.0%)
South	11 (8.40%)
Central	8 (6.11%)
North	6 (4.58%)
West	6 (4.58%)
Southwest	5 (3.82%)
Northeast	1 (0.763%)
Hospital level	
Tertiary (Grade III)	117 (89.3%)
Secondary (Grade II)	13 (9.92%)
Primary (Grade I)	1 (0.763%)
Hospital type	
General	108 (82.4%)
Specialized	22 (16.8%)
Community	1 (0.763%)
Implementation of vancomycin TDM	
TDM performed	78 (59.5%)
Trough-based TDM	64 (48.9%)
Peak and trough-based TDM	25 (19.1%)
AUC-based TDM	14 (10.7%)
TDM not performed	53 (40.5%)

Abbreviations: TDM, therapeutic drug monitoring; AUC, 24-h area under the curve.

### Implementation of vancomycin TDM

We investigated the overall implementation status of performing AUC-based TDM. Surprisingly, routine vancomycin TDM was administered in only 59.5% (78/131) of the surveyed hospitals. Moreover, only 10.7% (14/131) of these hospitals used AUC-based vancomycin TDM (Table 2). Of the eligible participants, 58.4% (94/161) had experience with vancomycin TDM, and only 37 participants (37/161, 23.0%) had the ability to estimate the AUC (Table 1). The hospitals surveyed preferred a combination of the two methods of monitoring (100/161, 62.1%), and more than half of the respondents indicated that they expected to conduct or transition to AUC-based monitoring within 1 year (92/161, 57.1%), although a significant number of respondents indicated that they were not sure about the need to transition (59/161, 36.6%).

### Perception about vancomycin TDM

The perceptions and knowledge of AUC-based monitoring in participants who had experience with vancomycin TDM are shown in Table 3. Participants identified patients at high risk of nephrotoxicity (74/94, 78.7%) as the preferred indications for vancomycin TDM, followed by critically ill patients (70/94, 74.5%). The most commonly accepted AUC/MIC target value for vancomycin was 400–600 (33/94, 35.1%), which was also recommended by American and Japanese guidelines. However, the appropriate AUC for vancomycin was still unclear for many people (45/94, 47.9%). In addition, participants considered the most appropriate vancomycin trough concentration targets to be 10–15 mg/L for adult patients (66/94, 70.2%) and 15–20 mg/L for adult patients with severe MRSA infections (64/94, 68.1%), which were recommended by the Chinese guidelines.

**TABLE 3:** Perceptions about implementation of vancomycin TDM.

Variable	Total (n = 94)
Indications for vancomycin TDM	
Patients at high risk of nephrotoxicity	74 (78.7%)
Critically ill patients	70 (74.5%)
Patients receiving high-dose vancomycin	64 (68.1%)
Patients with moderate to severe heart failure, or underweight patients	63 (67.0%)
Hemodynamically unstable patients	62 (66.0%)
Elderly patients (>65 years old)	62 (66.0%)
Pediatric patients, neonates	60 (63.8%)
Obese patients, burn patients	59 (62.8%)
Patients with augmented renal clearance	57 (60.6%)
Patients receiving prolonged courses of therapy (more than 3–5 days)	57 (60.6%)
Patients with MRSA infection	44 (46.8%)
All patients received vancomycin	35 (37.2%)
Others	2 (2.13%)
Vancomycin TDM target	
AUC/MIC	
400–600 in American and Japanese guidelines	33 (35.1%)
400–650 in Chinese and IATDMCT guidelines*	15 (16.0%)
Other or not sure	45 (47.9%)
Trough target	
2020 Chinese guideline	
10–15 mg/L in adult patients	66 (70.2%)
10–20 mg/L in patients with serious MRSA infections	20 (21.3%)
5–15 mg/L in pediatric patients or neonates	27 (28.7%)
2020 IATDMCT guideline	
10–15 mg/L in patients with serious MRSA infections	4 (4.26%)
2013 Japanese guideline and 2009 American guideline	
15–20 mg/L in patients with serious MRSA infections	64 (68.1%)
10–20 mg/L in adult patients	14 (14.9%)
10–20 mg/L in all infections	7 (7.45%)
Other	4 (4.26%)
AUC estimation method	
Can estimate AUC	37 (39.4%)
Bayesian modeling	33 (35.1%)
First-order PK equations with two concentrations	22 (23.4%)

Abbreviations: TDM, therapeutic drug monitoring; AUC, 24-h area under the curve; MIC, minimum inhibitory concentration; IATDMCT, International Association of Therapeutic Drug Monitoring and Clinical Toxicology; MRSA, methicillin-resistant *Staphylococcus aureus*; PK, pharmacokinetics. * Chinese and IATDMCT, guidelines suggested a AUC, target of 400–650 mg·h/L.

For the guidelines to change the monitoring index of vancomycin from the trough concentration to the AUC, pharmacists and physicians have varying perspectives. Of the 161 respondents, 35 pharmacists and physicians expressed their views on the current vancomycin TDM guidelines. Most of the respondents (24/35, 68.6%) supported that AUC monitoring is a more accurate and meaningful approach, which is highly conducive to individualized use in the clinic to improve therapeutic efficacy. However, a portion of the respondents (6/35, 17.1%) held a less optimistic view due to perceived complexities associated with AUC calculation and the current lack of sufficient high-quality evidence on benefits of AUC-based monitoring, thereby posing challenges for its routine implementation.

### Factors influencing the AUC-based vancomycin TDM implementation

**FIGURE 1:**
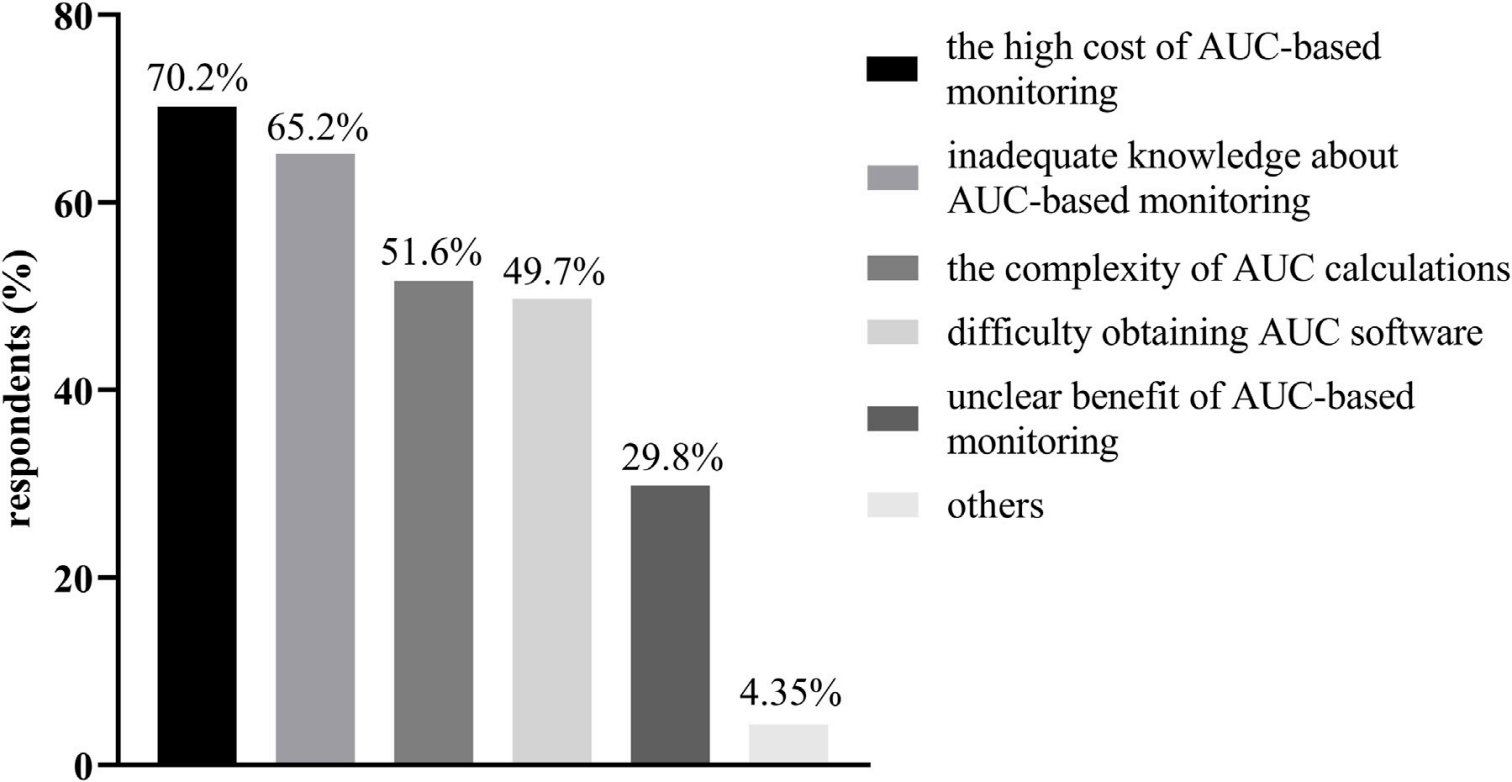
The challenges and barriers to implementing AUC-based monitoring as perceived by the participants.

The challenges and barriers to implementing AUC-based monitoring as perceived by the participants are shown in Figure 1. Unsurprisingly, the highest barrier to implementing vancomycin TDM was the cost of AUC-based monitoring (113/161, 70.2%), which included but was not limited to Bayesian software costs, and staff training costs. Inadequate knowledge about AUC-based monitoring (105/161, 65.2%) was the second challenge. The complexity of the AUC calculations and the difficulty of obtaining AUC software were also identified as important challenges by approximately half of the participants. Furthermore, the unclear benefit of AUC-based monitoring is also an important barrier that should be considered.

## Discussion

To the best of our knowledge, this is the first survey to evaluate the implementation status and perception of vancomycin AUC-based TDM in China. Our study included 131 hospitals from 20 provinces in China and could adequately reflect the status of vancomycin TDM. Based on the results of this study, vancomycin AUC-based TDM has not yet been widely implemented in clinical practice, and most hospitals still use trough-based TDM. The perceptions of pharmacists and physicians about vancomycin TDM were inconsistent with the current guidelines. Difficulties in AUC estimation and high cost were the main issues that needed to be accounted for before the implementation of AUC-based monitoring. It is too early to recommend AUC-based monitoring only in China, as well as other resource limited areas. This survey also demonstrated the dilemmas and doubts of vancomycin AUC monitoring, which may be helpful in its further implementation.

The revised vancomycin TDM guidelines, which recommend AUC-based monitoring, were published more than 3 years ago (He et al., 2020; Rybak et al., 2020; Matsumoto et al., 2022). However, this study revealed that AUC-based monitoring was still not commonly used among Chinese hospitals. Only 10.7% (14/131) of the responding hospitals adopted AUC-based monitoring, whereas 51.9% (68/131) used conventional peak and/or trough-based monitoring. Similar situations in other countries have also been reported. A cross-sectional survey of a national health consortium performed in 2019 showed that 23.1% of responding academic medical centers performed AUC-based TDM (Kufel et al., 2019). Another survey performed in 2022, 2 years after the publication of American updated guideline, revealed that only 29.7% of the institutions had implemented an AUC dosing program in hospitals across America (Bradley et al., 2021). It can be estimated that AUC-based monitoring is uncommon in developing countries. Thus, we can see that AUC-based monitoring only, as recommended by some guidelines, seems to be unsuitable for resource limited areas.

We also investigated respondents’ perceptions and knowledge of AUC-based vancomycin monitoring. It is concerning that guideline-recommended populations and TDM targets were inconsistent, which confused physicians and pharmacists. The Japanese guidelines recommend that AUC-guided TDM should be routinely used for all MRSA infections, irrespective of the severity or complexity of the infection (Matsumoto et al., 2022). Even in institutions where calculating the AUC using Bayesian methods is difficult, the use of AUC-guided dosing should be considered for patients at high risk of acute kidney injury (Matsumoto et al., 2022). Similarly, the guidelines published by the Anti-infectives Committee of the International Association of Therapeutic Drug Monitoring and Clinical Toxicology (IATDMCT) recommend that TDM should be indicated for all patients who are expected to receive vancomycin for longer than 48 h (Reuter et al., 2022). On the other hand, the guidelines published by the Chinese and American authors did not recommend vancomycin TDM for all patients but rather for patients at high risk of nephrotoxicity, patients with severe infections, neonates/children, and so on (He et al., 2020; Rybak et al., 2020). From the results we can see that respondents’ perceptions and knowledge of vancomycin were not fully consistent with any guidelines. Pharmacists and physicians were not able to timely track the updates of guidelines and deeper understand the changing of TDM targets. Therefore, it is paramount important to establish a more precise and clearer guidance for better clinical practice.

There is uncertainty in the academic community regarding whether AUC monitoring is required for all patients. In our previous study, we found that a trough concentration of 15–20 mg/mL had a good relationship with an AUC of 400–600 mg·h/L in critically ill patients not receiving renal replacement therapy, and trough-guide TDM may be sufficient in these populations (Yu et al., 2023). The other two studies proposed a similar idea. Huang et al. developed a hybrid model of trough and AUC monitoring through plan‒do‒study‒act (PDSA) cycles and reported that trough-based TDM was a pragmatic strategy for short-term anticipated dosing, while AUC-based TDM was the most impactful and cost-effective for patients at high risk of nephrotoxicity (Huang et al., 2021). The value of universal AUC-based monitoring was also questioned by Dilworth and Wright, who suggested that an easier and more effective way to reduce toxicity may be to focus on effective antibiotic stewardship to reduce overall prescribing rather than optimizing dosing based on limited hypothetical data (Dilworth et al., 2020; Wright et al., 2021). This evidence seems to indicate that AUC monitoring is not necessary for all patients. Therefore, high quality evidences are urgently needed for clinical decision making.

In addition, it is important to provide education or staff training to increase awareness of vancomycin TDM among pharmacists, physicians, nurses and laboratory staff, especially those using Bayesian software, to implement vancomycin TDM successfully. This education should provide personalized multimodal strategies with profession-specific content (Reuter et al., 2022). For example, physician education should focus on evidence or problem-based learning, while nurse education should include receiving clear instructions and protocols through in-service training (Van Dort et al., 2020). In contrast, for those who need to interpret the data to make dose recommendations, education based on the background and rationale for pharmacokinetics and pharmacodynamics should be provided to aid in understanding dosing decisions (Reuter et al., 2022). Furthermore, convincing studies about vancomycin TDM are needed to resolve these inconsistencies and achieve a consensus. We investigated the factors that impede the implementation of vancomycin AUC-based TDM. Unsurprisingly, participants generally identified monitoring costs as the most significant barrier. The annual cost of purchasing software, as well as subsequent software maintenance and staff training, may be enormous. However, a previous report showed that AUC monitoring was cost-neutral and could significantly reduce patient costs (Lee et al., 2020). However, this cost‒benefit study did not consider the impact of empirical therapies that are common in clinical practice or the implementation fees of EMRs and staff training; thus, the overall costs may have been underestimated (Lee et al., 2020). It is not surprising that the guidelines are more supportive of AUC-based dosing strategies than troughs are; this change would be an enormous task for hospitals, requiring significant time, effort, cost, and training (Bland et al., 2021). Therefore, we wanted to find a safe and feasible way to reduce costs and to accommodate the needs of medical institutions that are not equipped to conduct monitoring, for example, by establishing regional medical centers to centralize testing. Moreover, given that the majority of current models rely on sparsely sampled or limited datasets, a Bayesian-based vancomycin calculation website utilizing intensive sampling or a larger number of samples would significantly enhance AUC calculations. Additionally, implementing a decision tree model could effectively reduce unnecessary resource consumption.

Difficulties in the estimation of the AUC were one of the main barriers to the implementation of AUC-based monitoring. The guidelines recommend Bayesian estimation as the preferred method for calculating the AUC of vancomycin (He et al., 2020; Rybak et al., 2020; Matsumoto et al., 2022). Other methods, such as first-order PK equations, require two steady-state vancomycin concentrations, which may result in additional sampling and testing (Meng et al., 2019). Moreover, the calculation is complex. The advantage of Bayesian estimation is that the AUC of vancomycin can be estimated using trough-only data or plasma concentration data at any random time within the first 24–48 h (Rybak et al., 2020). Notably, the use of Bayesian software to calculate the vancomycin AUC and optimize the dose presupposes the use of a well-developed vancomycin population PK model as a Bayesian prior. Obviously, Bayesian programs adopting such priors are extremely rare, and most of them were developed based on sparse sampling (Aljutayli et al., 2022). On the other hand, there are differences in the clinical settings for which different software programs are applicable, so a combination of multiple software programs may be required to meet clinical needs (He et al., 2022). Furthermore, due to the heterogeneity among vancomycin population pharmacokinetic models, selecting an appropriate model for clinical use is not trivial. Models developed in a specific patient population may perform poorly when applied to more general inpatient populations or other patient populations, making them highly susceptible to bias in dosing decisions (Greppmair et al., 2023). Even for the same patient population, different models may lead to different results, which may be related to the sample size, heterogeneous study designs or assay methodology. Broeker et al. compared thirty-one published population pharmacokinetic models of vancomycin and elucidated that the relative bias and relative root mean squared error of the a priori predictions varied substantially (−122.7%–67.96% and 44.3%–136.8%, respectively) (Broeker et al., 2019). Therefore, some scholars recommend that extensive evaluation is required before applying any model to clinical patients (Guo et al., 2019).

Moreover, there is still uncertainty regarding whether the implementation of vancomycin AUC-based monitoring increases the likelihood of clinical cure. Systematic evaluation and meta-analysis revealed considerable heterogeneity in the pooled sensitivity and specificity of the vancomycin AUC/MIC ratio for predicting clinical outcomes, and the majority of these studies failed to demonstrate a relationship between the AUC/MIC and positive clinical outcomes (Dalton et al., 2020). Another retrospective study in patients with *enterococcal* infections showed that an AUC/MIC ≥400 was associated with significant differences in clinical and microbiological responses, as well as a higher rate of nephrotoxicity compared to an AUC/MIC <400 (Katip and Oberdorfer, 2021).

This study has several limitations. First, this electronic survey was widely distributed through social media (WeChat group), and we could not measure the true response rate because of the inability to know how many questionnaires were actually distributed; thus, it may introduce a non-response bias. Second, most of the hospitals surveyed in this study were tertiary care hospitals in Eastern China, and sampling bias may exist. In addition, some participants selected “other” for some questions and entered free text for clarification. The inclusion of these textual responses may still introduce bias, despite an independent review of these texts by our investigators. Furthermore, despite the considerable cost being the primary limiting factor for implementing AUC-based monitoring, we did not collect expenditure data comparing AUC-based and trough-based TDM. This aspect merits further investigation in future studies to enhance our comprehension of the feasibility of promoting AUC-guided TDM. Finally, we omitted collecting information regarding hospitals’ selection of software for calculating the AUC and evaluating its reliability. Such data could serve as a reference for other hospitals intending to conduct AUC TDM in the future.

## Conclusion

The majority of surveyed hospitals have not yet implemented AUC-based vancomycin TDM, especially in economically underdeveloped areas. The ability of physicians and pharmacists to estimate the AUC is also generally inadequate and requires further training. The highest ranked barrier to implementing vancomycin TDM was the cost of AUC-based monitoring, followed by the unfamiliarity of pharmacists and/or physicians. Given the low implementation rate and the lack of standardization of methods for estimating the AUC of vancomycin, it may be too early to recommend AUC-based TDM only, and trough-based monitoring is still needed. We look forward to more comprehensive analyses of vancomycin monitoring across diverse populations, and to developing a decision-tree model that will provide practical implementation strategies.

## Data Availability

The original contributions presented in the study are included in the article/Supplementary Material, further inquiries can be directed to the corresponding authors.
