# Supplementary material for: Is it time to recommend AUC-based vancomycin therapeutic drug monitoring only? A cross-sectional survey in China
Source: Front Pharmacol. 2024 Jul 12;15:1370040. doi: 10.3389/fphar.2024.1370040 (PMC11272526; doi:10.3389/fphar.2024.1370040)
Supplement: Supplementary file 1 [file DataSheet1.DOCX]

Table S1 The English version of the questionnaire.

Vancomycin has a narrow therapeutic window and large interindividual pharmacokinetic variability; thus, therapeutic drug monitoring (TDM) is needed. However, the vancomycin TDM guidelines recommend changing the vancomycin therapeutic drug monitoring target from trough to area under the concentration-to-time curve/minimum inhibitory concentration (AUC/MIC). We used a questionnaire to examine the implementation of vancomycin AUC-based TDM in China and the perceptions and knowledge of AUC-based vancomycin monitoring and to determine the difficulties and challenges in performing AUC-based TDM. The questionnaire will be used for research purposes only and will not be used for any other purpose, while your personal information will be kept strictly confidential. Thank you for your valuable time.

1. What provincial administrative regions of China is your hospital located in?
2. What is the name of your hospital?
3. Your hospital is:
4. Public hospitals
5. Private hospitals
6. Your hospital is:
7. Tertiary (Grade III)
8. Secondary (Grade II)
9. Primary (Grade I)
10. Your hospital is:
11. General
12. Specialized
13. Community
14. Your department is:
15. Your occupation is:
    1. Physician
    2. Pharmacist
16. What is your area of specialization? (multiple-choice question)
17. Respiratory
18. Infectious diseases
19. Intensive Care Unit (ICU)
20. Hematology
21. General
22. Other:
23. How many years have you been practicing?
24. 1-3 years
25. 4-6 years
26. 7-9 years
27. ≥10 years
28. Does your hospital conduct therapeutic drug monitoring (TDM) for vancomycin?
29. Yes
30. No (skip to question 22)
31. Who are the candidates for AUC-guided dosing？
32. All patients received vancomycin
33. Patients with MRSA infection
34. Critically ill patients
35. Obese patients, burn patients
36. Pediatric patients, neonates
37. Elderly patients (>65 years old)
38. Patients at high risk for nephrotoxicity, i.e., who are receiving continuous renal replacement therapy (CRRT), who are receiving concomitant nephrotoxic agents (for example, piperacillin/tazobactam, diuretics and so on), or who have impaired or unstable renal function;
39. Patients with augmented renal clearance (ARC)
40. Patients with moderate to severe heart failure or underweight patients
41. Patients receiving prolonged courses of therapy (more than 3 to 5 days)
42. Patients receiving high-dose vancomycin
43. Hemodynamically unstable patients
44. Other
45. Does your hospital perform vancomycin trough-based monitoring?
46. Yes
47. No
48. What is the appropriate vancomycin trough concentration goal?
49. 10-15 mg/L in patients with regular infections
50. 10-20 mg/L in patients with regular infections
51. 10-20 mg/L in patients with serious MRSA infections
52. 10-15 mg/L in patients with serious MRSA infections
53. 15-20 mg/L in patients with serious MRSA infections
54. 10-20 mg/L in all infections
55. 5-15 mg/L in pediatric patients or neonates
56. Other
57. Does your hospital perform vancomycin peak-based monitoring?
58. Yes
59. No
60. Does your hospital perform vancomycin peak and trough-based monitoring?
61. Yes
62. No
63. Does your hospital monitor vancomycin concentrations at other time points?
64. Yes
65. No
66. Does your hospital perform vancomycin AUC-based monitoring?
67. Yes
68. No
69. What is the appropriate vancomycin AUC target?
70. 400-600 mg·h·L^-1^
71. 400-650 mg·h·L^-1^
72. have not used AUC, not sure of appropriate targets
73. Other:
74. Do you have the ability to calculate vancomycin AUC via Bayesian methods?
75. Yes
76. No
77. Do you have the ability to calculate vancomycin AUC via a first-order PK equation with trough and peak concentrations?
78. Yes
79. No
80. Do you have the ability to calculate vancomycin AUC via a first-order PK equation with concentrations at other time points?
81. Yes
82. No
83. What type of monitoring would you prefer if vancomycin TDM were to be conducted?
84. Trough-based TDM
85. AUC-based TDM
86. Both of them
87. Do you expect to conduct or transition to the AUC-based monitoring within one year?
88. Yes
89. No
90. Not sure
91. What are the challenges or barriers to implementing vancomycin AUC-based monitoring? (multiple-choice question)
92. Inadequate knowledge of the AUC-based vancomycin TDM of pharmacists and/or physicians
93. High cost of AUC-based monitoring
94. Complexity of the AUC calculation
95. AUC software is difficult to use
96. unclear benefit of AUC-based monitoring
97. Other:
98. What are your suggestions or comments about changing the monitoring index of vancomycin from trough concentration to AUC?
